# Estimating treatment sensitivity in synthetic and in vitro tumors using a random differential equation model

**DOI:** 10.1038/s41540-025-00530-0

**Published:** 2025-05-23

**Authors:** Natalie Meacham, Erica M. Rutter

**Affiliations:** 1 Department of Applied Mathematics, University of California, Merced, Merced, CA USA; 2 Health Sciences Research Institute, University of California, Merced, Merced, CA USA

**Keywords:** Cancer, Applied mathematics, Computational science

## Abstract

Resistance to treatment, which comes from the heterogeneity of cell types within tumors, is a leading cause of poor treatment outcomes in cancer patients. Previous mathematical work modeling cancer over time has neither emphasized the relationship between cell heterogeneity and treatment resistance nor depicted heterogeneity with sufficient nuance. To respond to the need to depict a wide range of resistance levels, we develop a random differential equation model of tumor growth. Random differential equations are differential equations in which the parameters are random variables. In the inverse problem, we aim to recover the sensitivity to treatment as a probability mass function. This allows us to observe what proportions of cells exist at different sensitivity levels. After validating the method with synthetic data, we apply it to monoclonal and mixture cell population data of isogenic Ba/F3 murine cell lines to uncover each tumor’s levels of sensitivity to treatment as a probability mass function.

## Introduction

In the study of cancer, treatment resistance is a well-studied phenomenon for impactful reasons: How long a patient lives and their quality of life both depend on the efficacy of treatment. While there is a broad range of treatments available for a wide variety of cancers, it is relatively common for patients who have reached metastasis (the growth of malignancies away from the original location) to face resistance to conventional chemotherapy approaches. Even as newer classes of therapies emerge, resistant cancer remains a major cause of patient mortality^1^. Additionally, standard modes of treatment cause lower of quality of life for many patients^2^. It is clear that discovering different treatment approaches is necessary but insufficient for cancer patients—the scientific community must also focus on strategic applications of existing therapies to prevent and address resistance.

Scientists know the biochemical processes that control drug resistance^3–5^. However, treatment strategies based on that knowledge have still ended in treatment resistance^1,6^, and even newer strategies with more potential, like epigenetic drugs, require nuance and precision^7^. Saunders et al.^1^ claim that compared to the biochemical processes, intratumoral heterogeneity is the more fundamental driver of resistance. Specifically, cells within tumors vary genotypically or phenotypically, meaning they also vary in sensitivity to therapy. There are two models for how heterogenous tumors form: a cancer stem cell model, in which only a stem cell-like subpopulation can self-renew, and a clonal evolution model, in which most cell types can self-renew. Importantly, both models allow genotypically different clonal variants to grow in a way that creates a tumor with subpopulations that differ in sensitivity to treatment^1^.

There are a number of factors in both the drugs and the human body that contribute to drug resistance. Intratumoral heterogeneity, or the presence of multiple genetically distinct cell types within a single tumor, has been found to be a major driver^1^. Cancer treatment “selects for” resistance because some populations of cells are sensitive to a given treatment, while others are more resistant to that treatment. When the drug is applied, it kills the sensitive populations effectively, leaving behind the resistant cells, which then propagate, eventually building a tumor of cells that are largely immune to the treatment^8^. The mechanism through which resistant cells spread into the whole tumor space when sensitive cells are killed matches the ecological concept of competitive release^9^, wherein organisms like barnacles expand their populations into the space left behind when an adjacent species of barnacle is removed^10^. Competitive release addresses the idea that it is competition, rather than environmental factors like water depth, that keeps two different species of organisms in their respective parts of an ecosystem. Competitive release provides a framework for understanding why treatment resistance in cancer can develop when sensitive cells are no longer present to compete with resistant cells^11^.

Given how resistant tumors develop, it is key for clinicians to be able to adjust the treatment protocol as the disease and cell profile develop. In recent years, there has been an increase not only in mathematical cancer modeling but also in building tools to predict optimal treatment approaches^12^. Robert Gatenby pioneered one such approach with his research describing the possibility of curtailing cancer growth in the face of resistance to treatment. Using the analogy of invasive species management, where agriculturalists have found that intermittent insecticides applied only at some population threshold control invasive species without allowing insecticide-resistant strains, Gatenby advocates for cancer therapies that maintain a stable tumor mass rather than allowing resistance to develop by attempting eradication^8,13^. Adaptive therapy is a treatment protocol in which providers adapt treatment decisions as tumor dynamics evolve, motivated by optimizing survival without disease progression^8^. West et al.^14^ specify that adaptive therapy occurs in anticipation of, rather than as a consequence of, developing resistance in tumors. The primary difference between the current standard of care and adaptive therapy is that the current standard of care delivers a higher intensity of treatment with the goal of decreasing the tumor burden, without accounting for the potential development of resistance. Meanwhile, adaptive therapy maintains a higher tumor burden to allow sensitive and resistant populations to compete, delaying resistance^8,14,15^. As individualized medicine has entered mainstream medical dialog, adaptive therapy (and offshoots like range-bounded adaptive therapy^16^) has proven to be a valuable tool in tackling variations in cell heterogeneity and resistance for different patients^14^. In fact, for cancers like prostate cancer and recurrent high grade glioma, adaptive therapy is often more beneficial than continuous therapy^17–21^. Since adaptive therapy seeks to modify treatment plans before tumors evolve, meaning practitioners need to predict future outcomes based on limited data, there is a wealth of approaches mathematicians can take to contribute to advancements in adaptive therapy.

While cell heterogeneity has been established as a key component of resistance to treatment, the research is less conclusive on how to incorporate heterogeneity and resistance into mathematical models of tumor growth, especially in the context of work that hopes to support clinicians. For many types of cancer data, there is no method to measure what cell types occur in the tumor or how the populations may shift over time, meaning that there is no way to ascertain ahead of time when resistance may start to develop. Some research has access to multiple measurements from patients, such as MRIs and molecular profiling^22^, and other research tests the response of in vitro or ex vivo tumors to various drugs^23,24^, but that standard of data collection is rare and expensive in a clinical setting. Instead, there is often primarily aggregated data that depicts the volume of a tumor over time^21,25,26^, without information about what cell types constitute the tumor or how resistant to treatment they are. For oncologists choosing and administering therapies, more nuanced knowledge of a patient’s budding resistance can help inform more precise care with better outcomes. Thus, the motivation for this project is that using available aggregated tumor growth data to uncover developing resistance is key to maximizing patient well-being.

Given that cell heterogeneity has been confirmed as a key factor in how a patient responds to treatment, there is a clear directive for mathematical models to depict cell heterogeneity. Many models that incorporate heterogeneity consider only two or three separate cell types^27^, such as HER2+ and HER2- cells in breast cancer^28^, two generic cell types that can switch between types^29^, androgen dependent, androgen producing, and androgen independent cells in prostate cancer^18^, or malignant versus normal cells in two different environments^30^. Some works explicitly model heterogeneity in resistance to treatment as structured populations models^31–33^. For example, Greene et al. construct a structured population model in which the population is structured by time and also by level of resistance to treatment^32^. The ability to predict how a tumor will grow based on the resistance levels of different subpopulations is important, but it is unrealistic to know a priori how many subpopulations a given tumor has or how resistant to treatment each subpopulation is. Moreover, these types of model have not been parameterized with aggregated tumor volume data.

Because the reasons for resistance, levels of resistance, and number of cell types can vary so widely, it is necessary to develop a mathematical model that can handle nuanced subpopulations in a tumor. Furthermore, such a model would need to be parameterized with aggregate data. While not in the context of treatment resistance, previous models and frameworks have been developed that model and recover heterogeneity in growth and diffusion^34,35^. Rutter et al. address cell heterogeneity by depicting the growth and diffusion parameters as random variables in a random differential equation model^34^, resulting in a flexible model that does not need to predefine a small number of cell types. The framework developed in this work allows for recovery of the distributions of these random variables. Nguyen et al. extend the random differential equation model^34^ to account for competition between subpopulations and find that their random differential equation model predicts cell density more accurately than coupled partial differential equation models^35^, validating the concept of reflecting heterogeneity via random variables versus incorporating only a few subpopulations.

From the perspective of adaptive therapy, providers need regular updates on how a patient’s sensitivity to a given treatment is evolving^14^. There is a resulting need for mathematical models that can uncover a patient’s level of resistance given commonly available data, such as tumor size over time or similar biomarkers like prostate-specific antigen for prostate tumor size^36^. Furthermore, because a scalar value for resistance to treatment has limited value in the context of a heterogeneous tumor with multiple subpopulations, it is important to be able to recover as much nuance as possible about the level of resistance by recovering that information as a curve rather than as a single number. Kohn-Luque et al. provide a strong example of an approach to uncovering a nuanced picture of sensitivity to treatment, taking in cell count data from in vitro tumor samples and outputting both the frequencies and drug sensitivities of tumor subpopulations^37^. One primary drawback of ref. ^37^ is that it may not capture dispersal or variation in subpopulation growth rates, as it returns a scalar value for each subpopulation. We adapt the approach of refs. ^34,35^ to create a model that takes in tumor growth data and uncovers sensitivity to treatment as a probability mass function, allowing a detailed picture of how much resistance to treatment can be expected from the cell subpopulations within a given patient’s tumor. We develop the inverse problem methodology using synthetic data and validate on in vitro data of both monoclonal and mixture varieties^37^.

## Results

In this section, we present results from the mathematical model incorporating sensitivity as a random variable. We briefly explain the model here, but refer the reader to the methods section for a discussion on implementation. We propose describing the phenotypic heterogeneity by using parameter distributions for the parameter *s*, which represents the sensitivity to treatment. The simple model contains logistic growth and a log-kill term that is scaled by sensitivity value:

1 $$\frac{dc(t,{\bf{s}})}{dt}=\rho c(t,{\bf{s}})(1-c(t,{\bf{s}}))(1-{\bf{s}})-k{\bf{s}}c(t,{\bf{s}})$$

where *ρ* represents the maximal growth rate and *k* represents the death rate due to treatment (e.g., chemotherapy or radiation). In this work, we consider modeling treatment using the log-kill hypothesis^38^, which is still used in chemotherapy models today^39^. The assumption is that the parameter **s** is a random variable defined on some compact probability space Ω, in this case, [0,1]. To obtain the aggregated tumor volume over time, we calculate the expectation over that probability space using the probability measure *P*(**s**):

2 $$c(t)={\int}_{\Omega}c(t,{\bf{s}})dP({\bf{s}}).$$

We begin first by discussing results of the forward problem, in which the underlying distribution of sensitive and resistant cells is known, and the output is the projected aggregate tumor volume change over time. We follow this by presenting the results of the inverse problem via the Prohorov Metric Framework, which estimates *P*(**s**) (the distribution of sensitive and resistant cells) from aggregated tumor volume data under three increasingly challenging scenarios: synthetically generated tumors, monoclonal in vitro data, and two-population in vitro data.

**Fig. 1:**
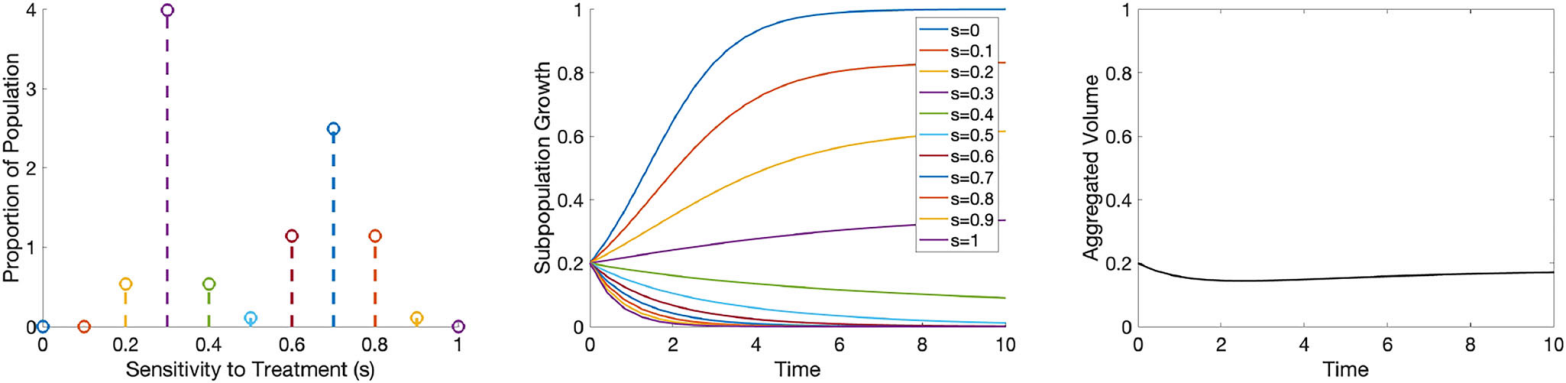
Schematic of the forward problem. For a sample bi-Gaussian distribution with 11 points (left), we solve Eq. (1) for each sensitivity value (middle) and find the weighted aggregate tumor volume using Eq. (2) (right).

In the forward problem, we start with a known distribution describing sensitivity to treatment over a specified mesh, which represents a given number of cell types with varying frequencies. See Fig. 1, left panel, for an example distribution in which the majority of cells have a low sensitivity to treatment and there is a small population of cells with a higher sensitivity to treatment. We solve Equation (6) at each sensitivity value to describe how each subpopulation grows independently over time (see Fig. 1, middle) and then take the weighted sum of those curves to obtain the curve depicting aggregated tumor volume (see Fig. 1, right panel). By successfully using a probability distribution to create a tumor growth curve that mimics patient data, we are able to lay the groundwork for the inverse problem described below.

### Methodology recovers sensitivity distributions for noiseless synthetic data

The central goal is to determine whether we can successfully estimate the distribution of sensitive and resistant cells (Fig. 1, left panel) given only the aggregated tumor volume data (Fig. 1, right panel). To validate the inverse problem methodology, we begin with synthetic data using known initial sensitivity distributions. We examine several different scenarios to test the methodology. First, we challenge the model with five different underlying distributions, including multiple continuous and discrete distributions. Second, we test the applicability of the methodology by assessing the ability to accurately recover distributions from sparse data. Finally, we test the robustness of the methodology by increasing levels of noise for each distribution.

The five distributions that we investigate are: (1) one-point distribution, (2) two-point distribution, (3) uniform distribution, (4) Gaussian distribution, and (5) bi-Gaussian distribution. These distributions are chosen to represent typical modeling scenarios, as well as to span both continuous and discrete options to test the flexibility of the inverse problem method. The one-point distribution simplifies to a single equation which does not account for treatment heterogeneity. The two-point distribution represents a modeling case in which one population is modeled as sensitive to treatment and the other is modeled as resistant to treatment. The normal and bi-Gaussian distributions represent one and two subpopulations, respectively, which include some variability around the mean(s). The uniform distribution was selected to assess code stability. The list below summarizes the distribution information for the underlying sensitivity values.

- One-point distribution: One point at *s*_1_ = 0.4 where *p*(*s*_1_) = 1.
- Two-point distribution: Two points at *s*_1_ = 0.3, *s*_2_ = 0.8 where *p*(*s*_1_) = 0.4, *p*(*s*_2_) = 0.6.
- Uniform distribution: Standard uniform distribution over 0 ≤ *s* ≤ 1.
- Gaussian distribution: Continuous Gaussian distribution with *μ* = 0.5 and *σ* = 0.09.
- Bi-Gaussian distribution: Continuous bi-Gaussian distribution composed of two Gaussians (*μ*_1_ = 0.3, *μ*_2_ = 0.7, *σ*_1_ = 0.05, *σ*_2_ = 0.1) that are summed and weighted with *w*_1_ = 0.5, *w*_2_ = 0.5.

It is possible for the normal and bi-Gaussian distributions to have nonzero values outside [0,1]. To combat this, we truncate the distributions and re-weight as:

$$p(s)=\left\{\begin{array}{ll}\frac{p(s)}{\mathop{\int}\nolimits_{0}^{1}p(s)ds}\quad &s\in [0,1]\\ 0\quad &s\,\notin\, [0,1].\end{array}\right.$$

This re-weighting ensures that all values are in [0,1] and the PDFs integrate to 1.

We note that in many cases, we are using a discrete PMF to approximate a continuous PDF. In these cases, the original and recovered distributions tend to have different scales. However, for clarity in visualization and interpretation, it is ideal to plot them on the same scale. For underlying discrete distributions, it is trivial to ensure identical scaling. For comparing recovered PMFs with underlying continuous PDFs, there are two main barriers to comparison: (1) the PMFs sum to 1 while the PDFs integrate to 1, and (2) there are likely a different number of nodes in the underlying and recovered distributions. We use the following scaling on the underlying continuous PDF to display the PMF and PDF on the same axis:

1. Normalize the PDF so that it adds to one: ${p}^{{\rm{new}}}=\frac{{p}^{{\rm{old}}}}{\sum {p}^{old}}$
2. Scale the new PDF by number of nodes in recovered PMF: ${p}^{{\rm{plot}}}={p}^{{\rm{new}}}\frac{{q}_{{\rm{PDF}}}}{{q}_{{\rm{PMF}}}}$

where *q*_PMF_ and *q*_PDF_ are the number of nodes for the recovered PMF and underlying PDF, respectively. We note that this transformation results in the continuous PMF neither summing nor integrating to 1. While this adjustment provides a *visualization* that is on the same scaling, it does not allow us to quantitatively compare the PDF and PMF due to the differing numbers of nodes in the underlying and recovered distributions. The unedited original recovered distributions for noise-less synthetic data are contained in Supplementary Fig. 3.

**Fig. 2:**
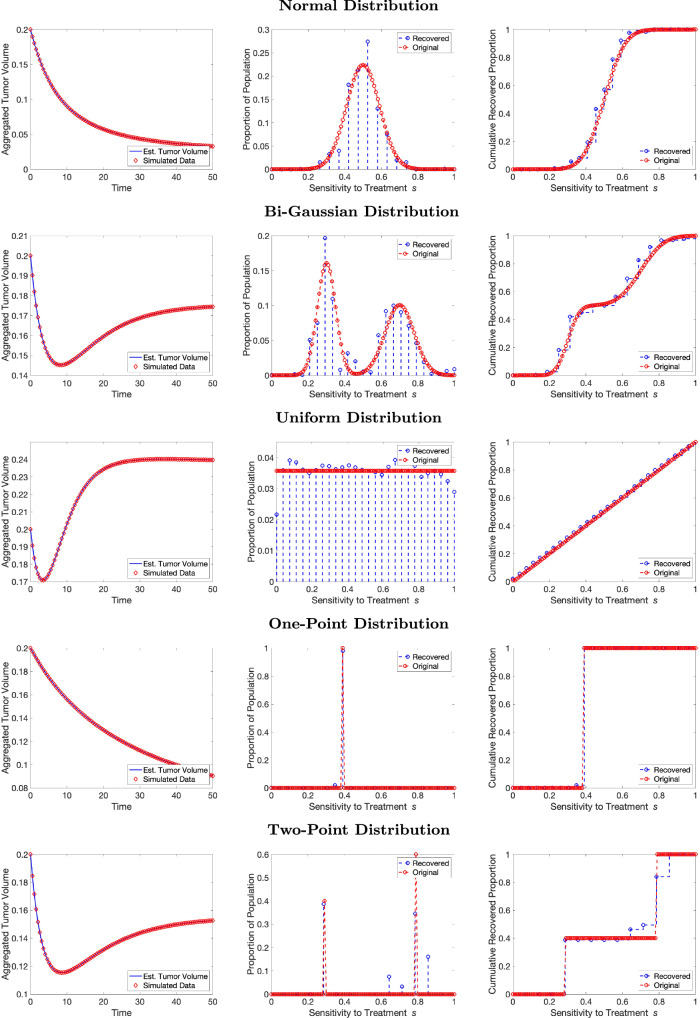
Recovered sensitivity distributions. Model fit to the synthetic population data (left), recovered PMFs (center) and recovered CDFs (right). The synthetic data is in red and the model fits are in blue. Each row represents a different underlying distribution.

Figure 2 displays the results of our first experiment testing the five different underlying distributions in the absence of noise. Each row represents a different distribution, with the first row the normal distribution, the second row the bi-Gaussian distribution, the third row the uniform distribution, the fourth row the one-point distribution, and the fifth row the two-point distribution. In the leftmost figures, the synthetic data (red diamonds) is depicted alongside the aggregate tumor volume curve generated by the recovered PMF (blue line). We observe that for all synthetic data distributions, the aggregated tumor volume is consistently a very strong fit. The middle figures depict the original underlying PMF or PDF (red line) alongside the recovered PMF (blue line with points). Because the original and recovered distributions tend to have different scales due to their different meshes in the case of continuous original distributions, the continuous original distributions have been scaled to use the same axes as the recovered discrete PMFs. For underlying distributions that are discrete (one-point and two-point), the scales are the same for the recovered and original distributions. The rightmost figures depict the cumulative probability density functions for both the original (red line) and recovered (blue line) distributions.

We observe that for both the continuous and discrete distributions, we are able to fit tumor growth curves well (left panels). When looking at the recovered distributions, we see that the method successfully recovers a probability distribution that reflects the shape of the original PMF (middle panels). In particular, we obtain very good estimates for the normal distribution (Fig. 2 top middle) and one-point distributions (Fig. 2, fourth row middle). The uniform (Fig. 2, third row middle), two-point (Fig. 2, bottom row middle), and bi-Gaussian (Fig. 2, second row middle) distributions are also relatively well-recovered. The selected distributions that had multiple subpopulations (two-point and bi-Gaussian) had relatively equal weights between subpopulations. To test whether the method is successful for more imbalanced distributions, we also tested a suite of bi-Gaussian distributions, including some that had weights of 10% and 90% for the subpopulations, respectively. We show good ability to recover these distributions even when there is a smaller subpopulation (see Supplementary Fig. 1 and Supplementary Table 1).

### Testing the method with sparse and noisy synthetic data

Clinical data often has sparse time resolution due to expense or difficulty in collecting data. Therefore, for our method to be clinically relevant or applicable, it is paramount that the distribution recovery be robust with respect to sparsely collected data. The recovered distributions displayed in Fig. 2 assume that 100 time points worth of data is available, which is unrealistic. Here, we test the ability to recover the underlying distributions under decreasing amounts of data.

We note that for clarity in visualization, we re-scaled the learned PMFs for the continuous distributions, as described in previous section. We consider ${q}_{{N}_{t}}$ to represent the number of nodes recovered in the distribution for *N*_*t*_ time points (e.g., *N*_*t*_ = 5, 10, 25, 100). We select the maximum of those four values (${q}_{{N}_{{t}_{\max }}}$) to represent the default *y*-axis limits. The remaining PMFs are scaled by

$${p}_{{\rm{scaled}}}({s}_{j})=p({s}_{j})\frac{{q}_{{N}_{t}}}{{q}_{{N}_{{t}_{\max }}}}.$$

This scaling allows for the recovered PMFs to be plotted roughly on the same *y*-axis limits and directly compared to one another. However, this scaling also means that the plotted PMFs may not add to one.

**Fig. 3:**
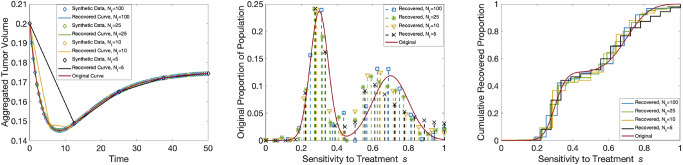
Sparse data recovery. Model fit to the synthetic population data (left), recovered PMFs (center) and recovered CDFs (right) for an underlying bi-Gaussian distribution (red) for 100 (blue), 25 (green), 10 (yellow), and 5 (black) data points.

Figure 3 displays the resulting fits (left), recovered PMFs (middle), and CDFs (right) for the bi-Gaussian distribution with 100 (blue squares), 25 (green asterisks), 10 time points (yellow triangles), and 5 time points (black x’s) compared to the true underlying distribution (red line). It is clear that the method is still able to appropriately capture the main features of the recovered distribution even with as few as five time points. While we have only shown the recovery for the bi-Gaussian distribution here, similar results hold for the remaining distributions. Comparable figures for the remaining distributions are located in Supplementary Fig. 4.

**Fig. 4:**
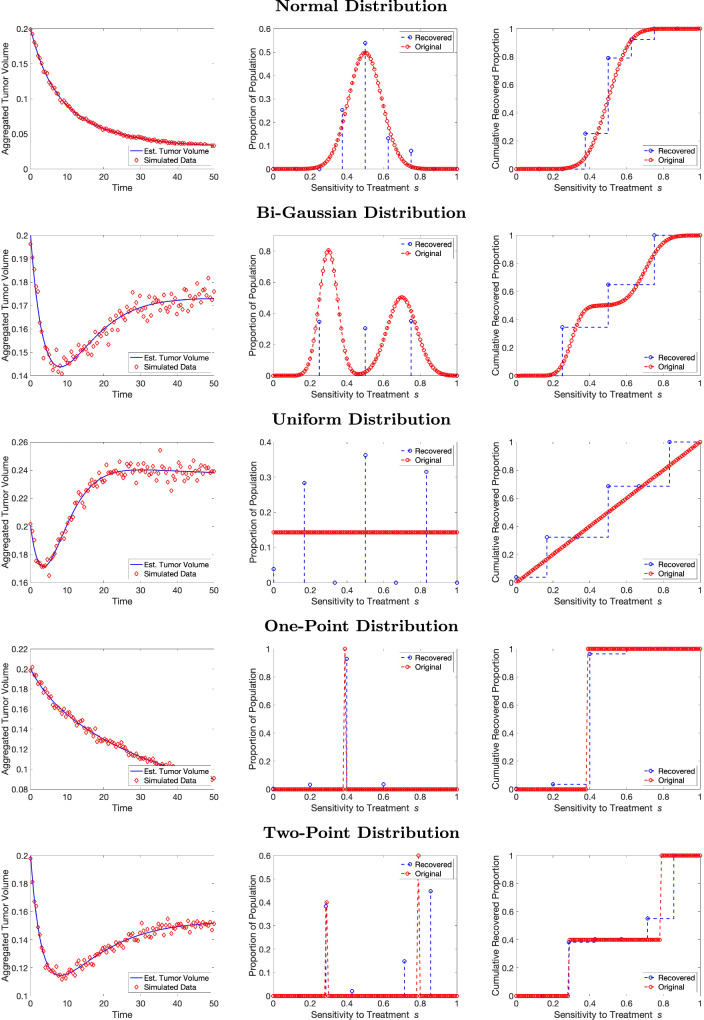
Noisy data recovery. Results for synthetic data for five different distributions under 2% noise. Model fit to the synthetic population data (left), recovered PMFs (center) and recovered CDFs (right). The synthetic data is in red and the model fits are in blue.

To assess the robustness of the method, we add proportional error noise up to 5% and examine the ability to fit the data and recover the distributions. Figure 4 displays the fits for all five distributions with 2% noise added to the solutions. As with Fig. 2, each row represents a different underlying distribution: the left panels show the fit to the total tumor volume, the middle panels compare the original and recovered PMFs or PDFs, and the right panels depict the recovered CDFs. Please see Supplementary Figs. 5 and 6 for fits to all five distributions with 1% noise and 5% noise, respectively.

When examining Fig. 4, we observe that even with added noise, the method is able to recover discrete distributions (e.g., the one-point and two-point distributions) quite well. Both the one-point and two-point discrete distributions, which biologically symbolize tumors with one or two cell types, respectively, have close fits to the data. Additionally, the inverse problem recovers the location of the points in the distribution well. However, the recovery of continuous underlying distributions does not perform as well in the presence of noise. The continuous uniform distribution, which biologically symbolizes the unlikely occurrence of a tumor’s subpopulations smoothly varying between equally likely probabilities, has visually the worst results, as the fit to the data is still very strong but the recovered PMF strays far from the original shape. The other two continuous distributions, the Gaussian and bi-Gaussian, which biologically represent smoothly varying cell types with probability clustered around one or two means, obtain strong fits to the data. The Gaussian returns a strongly matched probability distribution as well. However, the bi-Gaussian loses the detailed shape of the recovered PMF found under less noisy conditions.

From these results, we can draw a few conclusions. Firstly, in all cases, we are able to fit the total tumor volume data quite well. Secondly, we are able to recover the discrete underlying distributions with accuracy even with noise added. Thirdly, despite the good fit to the data, the recovered distributions for underlying continuous distributions do not obtain as strong of a fit. We hypothesize there could be several reasons for the lower-quality continuous results. One is that we are using the AIC to select the best fit model. It appears that in the presence of noise, a minimal number of nodes is always selected as the best fit (usually fewer than 8 nodes). It is possible that AIC might not be the best option for selecting the optimal model. At the low end of the numerical mesh (4 parameters) the regular AIC is applicable, but at the high end of the numerical mesh (30 parameters), the corrected AIC may be more relevant^40^. A second possibility is that using discrete approximations to a continuous function is not sufficient in the face of noise. Alternative methods for recovering a continuous distribution using spline functions have been previously developed^34,41^ and may yield better results.

### Description of in vitro data

While the framework has been validated on synthetic data, it is imperative to test its ability to work on real-world data. In this section, we will challenge the method with multiple sets of in vitro data from high-throughput drug screening experiments^37^. Below we briefly discuss the two different datasets used (monoclonal and mixture) followed by the resulting parameter estimation.

Kohn-Luque et al.^37^ provide several types of tumor growth data applicable to the model. We briefly summarize the relevant data description here but encourage readers to read the manuscript for full details^37^. There are four populations of monocolonal isogenic Ba/F3 murine cell lines, two transformed with the BCR-ABL fusion oncogene and two made resistant to the cancer growth blocker imatinib by the BCR-ABL-T3151 mutation. The two sensitive datasets were formed with approximate initial cell counts of 500 and 1000, respectively, creating datasets S500 and S1000, and the two resistant datasets were formed with approximate initial cell counts of 250 and 500, respectively, creating datasets R250 and R500. For each dataset, each monopopulation was treated with imatinib at 11 different concentrations (see Table 1 for the drug concentration values). The tumor cell density was measured at 14 time points (three-hour intervals between 9 and 48 hours). Each concentration has seven biological replicates.

**Table 1:** Dosage amounts for each concentration of drug provided in ref. ^37^

Dose #	1	2	3	4	5	6	7	8	9	10	11
(*μ*M)	0	0.0260	0.0536	0.1094	0.2244	0.3410	0.4603	1.1908	2.4430	3.7133	5.0119

A visual representation of the values is available in Supplementary Figure 7.

To prepare the data for use in the inverse problem, we average across the seven replicates for each concentration while disregarding any NaN values. Moreover, because the model in Equation (6) is scaled for a maximum population of one, we scale the data by choosing ten times the maximum cell count across all replicates, dosages, and time points of all the sensitive and resistant datasets to be the maximum threshold for cell count. Hence, the data fed into the model is averaged across the biological replicates and scaled. The initial population given to the model is the scaled population from the averaged data at the first time point.

In order to accurately estimate the distributions of sensitive and resistant cells, it is imperative to have a good estimate on the maximal growth rate of cells as well as the maximal death rate of cells under treatment. The maximal growth rate of cells under treatment is obtained by fitting a logistic equation to the resistant population in the absence of drug (dose 1). The maximal death rate of cells under treatment is obtained by fitting a decaying exponential equation to the sensitive population with the highest drug concentration applied (dose 11). We then linearly scale the death rate by the dosage concentration (see the “Methods” section for more details). In this model, we make the assumption that when cells are *fully resistant*, they do not die due to treatment, and their growth is not affected by increasing drug dosage. In contrast, when cells are *fully sensitive*, we assume that they do not grow, and that their death rate scales with the dosage concentration.

As described by ref. ^37^, the mixture data is created by performing a similar process to the one that generates the monoclonal data, but the resistant and sensitive populations are mixed together in four different proportions: 1:1, 2:1, 1:2, and 4:1 parts sensitive cells to resistant cells. Additionally, each experiment uses 14 biological replicates for each proportion rather than seven. We prepare the data for the inverse problem in the exact same manner as we do for the monoclonal data, including using the same maximum cell count for scaling.

### Method distinguishes between more and less sensitive monoclonal datasets

**Fig. 5:**
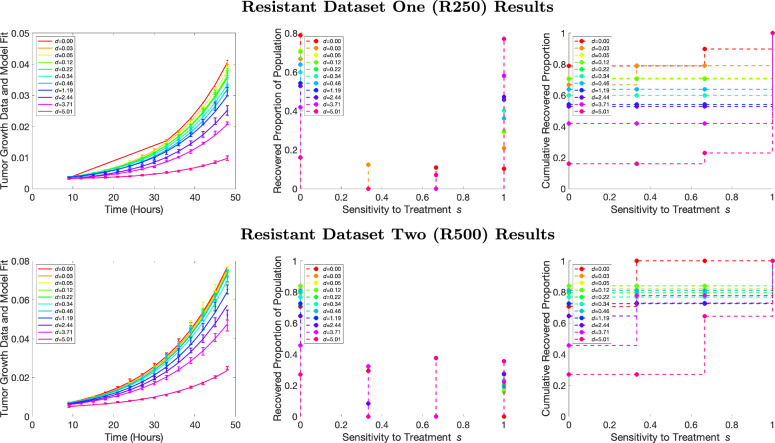
Recovered sensitivity distributions for resistant monoclonal data. Model fit to the resistant population data for all eleven concentrations (left), recovered PMFs (center), and recovered CDFs (right). Dosage concentrations are lowest for red and highest for pink. The top row of figures represents a resistant dataset initialized with fewer cells than the bottom dataset.

**Fig. 6:**
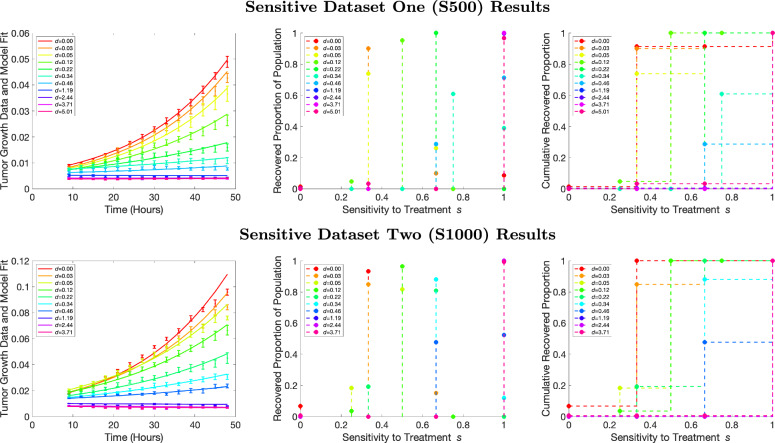
Recovered sensitivity distributions for sensitive monoclonal data. Model fit to the sensitive population data for all eleven concentrations (left), recovered PMFs (center), and recovered CDFs (right). Dosage concentrations are lowest for red and highest for pink. The top row of figures represents a sensitive dataset initialized with fewer cells than the bottom dataset.

Figures 5 and 6 display the results of our model for the resistant and sensitive monoclonal datasets from ref. ^37^, respectively. For each figure, the left panels represent the model fit to the aggregated tumor volume, while the center panel represents the recovered PMF and the right hand panel represents the recovered CDF. Each sensitive or resistant category of dataset contains two different initial cell counts as described above, so the top row corresponds to the smaller initial cell count monoclonal dataset while the bottom row corresponds to the higher initial cell count monoclonal dataset. Within each figure, the tumor volumes over time for each of the eleven drug concentrations are plotted (lowest concentration in red, highest concentration in pink).

Figure 5 depicts the fit and recovered distributions for the resistant monoclonal data. Note that the data, visualized with error bars on the left panels, have varying rates of growth depending on the drug concentration applied. While the raw resistant data reaches much higher cell counts than the raw sensitive data for the same initial cell count, indicating resistance, the dependence on dosage means that the resistant data is not fully resistant, given our mathematical definition above. However, since the cells are largely resistant, we would expect our recovered distribution to heavily favor sensitivity values near 0. The model is optimized to fit the aggregated tumor concentration (Fig. 5, left panel). The optimized model fits the data relatively well for all drug concentrations. The recovered PMF distributions (Fig. 5, middle panel) are largely predicted to be insensitive (or resistant) to treatment for the lower dosages, which matches with our biological knowledge of the dataset. However, for the higher dosages, the model recovers more sensitivity than resistance to treatment, with the highest dosage in pink being described as almost completely sensitive to treatment for R250. When examining the fit for that highest drug dosage (left panel, pink curve), it is clear that tumor growth is largely suppressed under treatment.

Figure 6 depicts the results for the sensitive monoclonal data. Similar to how the resistant data is not uniformly resistant to treatment, the raw sensitive data unquestionably grows much less than the raw resistant data, but the tumor growth does vary with dosage. Importantly, the sensitive cells shrink in number only for the two strongest doses, while some growth continues for smaller doses. Given our biological knowledge, we would expect the recovered distributions to be clustered towards higher sensitivity values. The model matches the tumor growth data closely for each drug concentration (leftmost panel). However, when looking towards the recovered PMFs, (middle panel), we do not see the expected behavior of recovered distributions clustered around sensitivity values close to one. Instead, we see that the sensitivity value scales with the drug dosage concentrations. The recovered PMFs for the lower drug concentrations hover around sensitivity value of 0.3 and then increase to sensitivity of 1 at the highest drug concentrations. The shape of each recovered PMF tends to favor the smallest numerical mesh possible, indicating that the best fits feature only a few subpopulations. The sensitivity values at the peaks of the recovered PMFs shift as the drug concentration increases or decreases, and so we observe that lower drug concentrations correspond to less sensitive populations and higher drug concentrations correspond to more sensitive populations.

There are some possible reasons why the recovered PMFs are not what we might expect. Firstly, a main assumption of our model is that subpopulations labeled “sensitive” are *fully* sensitive to treatment. When subpopulations are fully sensitive, the model simplifies to

$$\frac{dc}{dt}=-kc$$

so there is no opportunity for cell growth, only decay. When examining the data, it is clear that for lower dosages, the tumor is still growing. However, at high enough dosages, we observe that the data does indeed decay. Secondly, given the dependence of the PMF on the dosage concentration, we hypothesize that the way in which we are scaling the death rate due to different dosages might be incorrect. In brief, we *linearly* scale the death rate from the minimal death rate (at the lowest concentration) to the maximal death rate (at the highest concentration). It is possible that this scaling is not sufficient to capture the true dynamics of increasing dosage. Our mathematical assumptions for a label of full resistance are that there is zero effect on growth in the presence of drug, no matter the concentration. Therefore, looking at the resistant cell line data, in which tumor growth is still suppressed under treatment, it is perhaps not so surprising that we do not recover full resistance. For example, when examining the resistant dataset (left panel Fig. 5), it does appear that the cell line is largely resistant to treatment, until the dosages are very high. Thus, linear scaling may not hold. For the lower dosages, we do see the expected result of high amounts of predicted resistant cell population. However, both groups were given the same 11 drug concentrations^37^, but the sensitive populations’ recovered PMFs cluster around higher sensitivity values than the resistant populations’ recovered PMFs. Similarly, the CDFs from the sensitive populations add up to one more slowly. Hence, the model is still able to clearly distinguish between the two different cell types even if sensitive cells are not labeled *s* = 1 and resistant cells are not labeled *s* = 0. Future work could involve simultaneous fitting of all 11 drug concentrations jointly (since they should all exhibit the same levels of sensitivity) while simultaneously fitting the maximal growth rate and maximal death rate due to treatment.

### A mixed bag in sensitivity recovery for in vitro mixture datasets

**Fig. 7:**
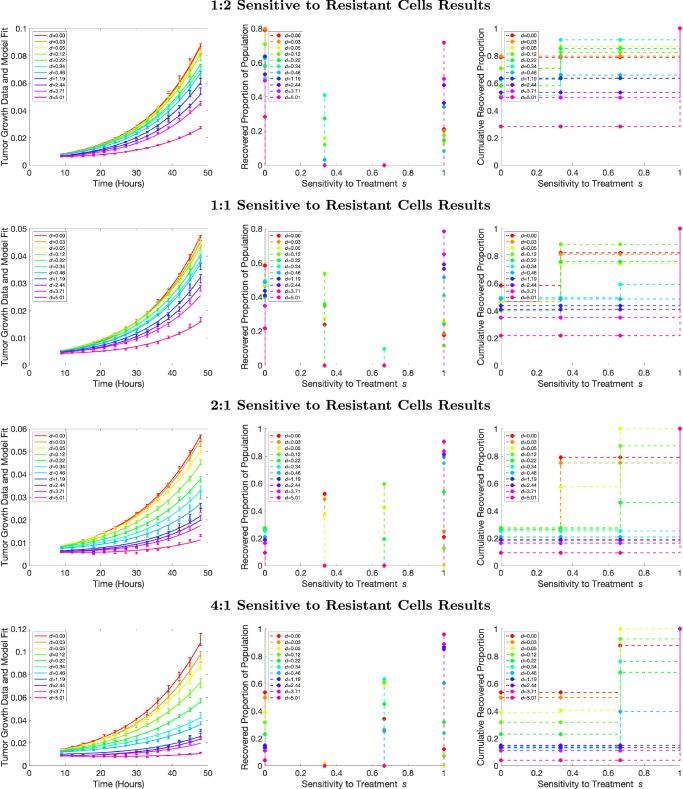
Recovered sensitivity distributions for mixture data. Model fit to the resistant population data for all eleven concentrations (left), recovered PMFs (center), and recovered CDFs (right). Dosage concentrations are lowest for red and highest for pink. The top row represents a true mixture ratio of 1:2, the second row represents a true mixture ratio of 1:1, the third row represents a mixture ratio of 2:1, and the bottom row represents mixture ratio of 4:1 sensitive to resistant cells.

Figure 7 depicts the inverse problem results for each of the four mixture datasets, with the rows representing the results from the 1:2, 1:1, 2:1, and 4:1 ratios of sensitive to resistant cells, respectively. As with the monoclonal results, the left-hand panel displays the aggregate tumor volume data compared with our model fits, the center panel displays the recovered PMFs, and the right panel displays the resulting CDFs. The leftmost column for all rows indicates that the overall fits to the data are reasonable for each of the drug concentrations applied.

When analyzing the recovered PMFs, we first should discuss the expectations. Since we know that two cell lines were mixed with varying initial concentrations, we would expect our recovered PMFs to have a cluster around 0 sensitivity, representing the resistant strain, and a second cluster around sensitivity values of 1, representing the sensitive strain. When examining the recovered PMFs, it is clear that the model is distinguishing between the different ratios of cell types, with the sensitivity values in the distributions sitting primarily at zero (fully resistant) and one (fully sensitive) in different proportions. If we examine dosages separately, we notice that as the known ratio of sensitive cells gets higher, larger proportions of the population have a sensitivity value of one or close to one. These results show that the model can satisfactorily distinguish between different proportions of sensitive and resistant cells at a specified dosage value.

While the model is able to depict the presence of two subpopulations, we do note that the proportions of sensitive and resistant cells are not always reflective of the known proportions. For example, the second row should be a mix of 1:1 sensitive and resistant cells. Therefore, we would expect a recovered proportion of population to be 0.5 at both sensitivity values of 0 and 1. Our fits show a wide range of resistant proportions, ranging from about 0.2 (for the highest dosage) to 0.6 (for the lowest dosage). We hypothesize that the same issue with drug dosage scaling that affected the sensitive monoclonal recovery is present in the mixture recovery as well. To investigate this hypothesis, we examine how the recovered distributions are affected by dosage. Specifically, we calculate the mean recovered sensitivity value for each of the monoclonal and mixture datasets and plot it against the dosage value. We define the mean recovered sensitivity value as:

3 $${\mu }_{{\bf{s}}}=\mathop{\sum }\limits_{j=1}^{q}{s}_{j}p({s}_{j}),$$

where *q* is the number of points in the recovered distribution, *s*_*j*_ is each sensitivity value, and *p*(*s*_*j*_) is the probability of that sensitivity value. We would expect that each dataset would have a flat mean sensitivity across dosage – that is, the mean sensitivity value should not be impacted by increasing dosage. Moreover, we would expect that the mean sensitivity values would be smaller for more resistant datasets and higher for more sensitive datasets.

**Fig. 8:**
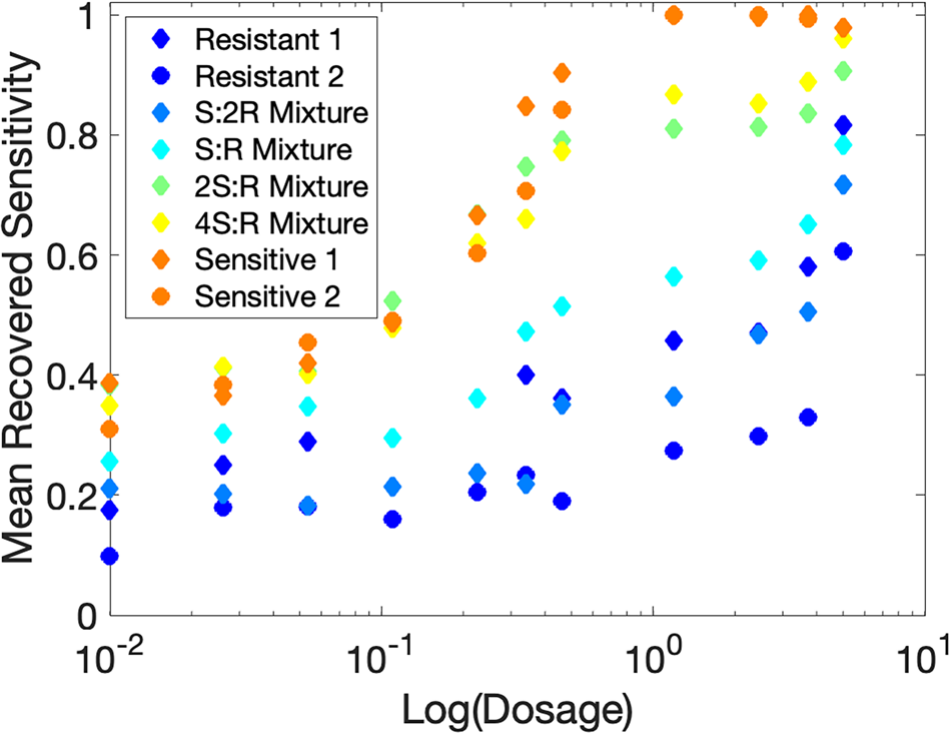
Mean recovered sensitivity versus drug dosage. For each dosage in *μ*M and each of the eight datasets, the mean recovered sensitivity is calculated using Eq. (3). Although the ordering at each dosage value is correct, we notice that mean sensitivity increases as dosage increases. The zero dose is plotted at 0.01 to allow log-scaling.

Figure 8 depicts the mean sensitivity value for the inferred distributions for all monoclonal and mixture datasets against the drug dosage value. Please note that the zero dose was rescaled to 10^−^^2^ to enable plot dosages on a log scale. Our first expectation is that mean sensitivity values should be lower for more resistant datasets and higher for more sensitive datasets. In the figure, it is clear that the monoclonal resistant datasets do show the lowest mean sensitivity (dark blue) and that monclonal sensitive cells show the highest mean sensitivity (orange). The various mixture populations lie in between. However, our second expectation is that the mean sensitivity value does not change with dosage value. In this case, it is clear there is a relationship between mean sensitivity and dosage. For the more sensitive populations, there is a positive correlation, approximately in the shape of an S-curve, between dosage and mean sensitivity, because the data that corresponds to high dosages results in inferred higher sensitivity. The resistant datasets also have a positive correlation between dosage and mean sensitivity, shaped like linear or possibly exponential growth. We hypothesize this may be due to misspecification in how we linearly scale death rates by the dosage value. As expected, the mean recovered sensitivity for the mixture datasets sits between the results for the sensitive and resistant datasets.

## Discussion

In this manuscript, we introduced a method to estimate the distribution of sensitive and resistant cells in a cancer growth and treatment model. The mathematical model is a random differential equation model of tumor growth and treatment in which sensitivity to treatment is expressed as a random variable with some underlying distribution. We showed the ability of the inverse problem to accurately recover sensitivity distributions for a wide range of distributions in the context of synthetic data, including with fewer time points and added noise. Finally, we challenged the model with in vitro monoclonal and mixture data^37^.

With the synthetic data, we were able to match the curve depicting tumor size over time, determine how sensitive to treatment the tumor is, and additionally confirm the subpopulation structure. In this study, we have shown the ability to recover underlying treatment resistance. Our nominal results featured synthetic data that was highly temporally resolved. The ability to accurately reconstruct subpopulation composition with as few time points as possible is paramount to the success of this method, as clinical data is typically not highly resolved. Our results showed that with as few as five data points, we were still able to recover the distribution of treatment resistance. However, whether or not five time points is realistic in clinical practice likely depends on the type of cancer being investigated. On the other hand, if high-throughput screening assays like the in vitro data generated in ref. ^37^ become more common in practice, this method appears to be robust enough to recover distributions with that temporal resolution. Future work will investigate the ability of this method to perform with in vivo data. When testing the method with added noise, we were able to show good fits to the tumor volume data, but the recovered distributions were not as accurate as the noiseless case, especially for continuous underlying distributions. We hypothesize that implementing spline-based approximations^34,41^ may prove more successful for recovery of underlying continuous distributions.

After validating the method on synthetic data, we then tested the method on previously published in vitro data^37^. In general, we were able to fit the tumor growth curves well. However, the recovered distributions were a mixed bag. When examining the mean sensitivity of the recovered distributions, the method was able to correctly order experiments from least to most sensitive. On the other hand, the mean sensitivities were also scaled by dosage amount, appearing more sensitive as drug dosages increased. For example, while the resistant data returns PMFs that are almost completely at *s* = 0 for lower dosages and higher sensitivity values for higher dosages, the sensitive data returns a more varied set of PMFs that show peaks from around *s* = 0.3 to *s* = 1, scaling with the increasing dosages. Because the sensitive data itself shows significant growth for several of the lower drug concentrations, mirroring the shape of some of the resistant curves, it makes sense that the model interprets that tumor growth data as coming from a cell type that is not fully sensitive to treatment. To circumvent this issue, future work may consider estimating the growth rate *ρ* and the death rate *d* directly, instead of estimating the sensitivity parameter *s* (as in refs. ^34,35^).

Although the model can be readily applied to patient data, some of which is available in ref. ^37^, there are relevant questions about how to address the differences between in vitro data and in vivo data. First, data taken from human subjects may involve fewer data points or more missing data, or different errors in terms of how tumor size or cell count is measured. Second, with little or no prior information about how sensitive to treatment a given patient is or what the natural carrying capacity for the tumor is, there may be additional challenges in scaling the growth and death rates. Additionally, it is not clear how many subpopulations with different sensitivities exist in real-world in vivo data, although^37^ estimated two subpopulations of differing treatment efficacies in multiple myeloma patient data. Despite such questions, it is a natural extension to use the model to fit in vivo patient data. One of the most useful benefits of our approach is not necessarily that we can determine the number of subpopulations, but rather that it can be used to see how the distribution of subpopulations is changing in response to treatment. For example, in the context of treatment methods involving multiple treatment windows, such as for prostate cancer^36^ or in the context of adaptive therapy^14^, we expect that this model could be used to investigate how the sensitivity level changes over treatment cycles. This could help predict whether additional cycles of therapy would be useful or whether the patient is developing resistance. Moreover, this approach could be used to test, in silico, how to optimally structure treatment dosing and schedule to delay the emergence of resistance^42–44^.

There are several limitations for both the in vitro results and the model formulation. In the context of the in vitro data, without prior knowledge of factors like the size of the dish where the cells are grown, it is difficult to find the carrying capacity for a given tumor. As such, the monoclonal and mixture datasets are scaled according to the single maximum cell count across all monoclonal datasets. Hence, while the normalization of the data does not significantly affect the accuracy of the model fitting or parameter estimation, it is still an estimate. Future work may consider jointly estimating the sensitivity parameter distribution *s* alongside the static parameter for carrying capacity. One limitation of the model is that in its current state, cell subpopulations do not compete directly with each other, but competition between subtypes is a necessary component of developing resistance to treatment^8^. A future development of this model formulation would be to introduce subpopulation competition as in ref. ^35^.

To ensure that this method is applicable in clinical settings, sensitivity analysis, uncertainty quantification, and parameter identifiability of the method need to be investigated^17^. In particular, it is imperative to understand under which data conditions we can reasonably expect to accurately recover parameter distributions. There are two types of identifiability that we would ideally want to investigate: structural and practical. Structural identifiability refers to the ability to uniquely identify the parameters under the assumption of "perfect data", and is inherent in the model structure. Practical identifiability considers whether or not we can uniquely determine the parameters given a specific dataset. In this manuscript, we interrogated the system with modest amounts of noise (2%) as well as with as few as five time points and were able to recover subpopulations reasonably well. However, a full identifiability analysis is more complicated due to the nature of the method. Specifically, due to the constraints of the system (all parameters must sum to one), the parameters are inherently dependent upon each other. Therefore, a natural future direction is to develop parameter identifiability for this method.

## Methods

### Forward problem

To describe how tumor volume changes over time while incorporating cellular heterogeneity with respect to treatment, we create a random differential equation depicting cell density over time, with sensitivity to treatment as a random variable. We briefly summarize the model introduced in Eqs. (1)–(2) and then discuss the discretization for computation. The model consists of tumor growth and treatment-induced death, where both terms are scaled by the sensitivity parameter **s**, a random variable. To calculate the total tumor volume at time *t*, we take the expected value over the compact probability space Ω (in our case, Ω = [0, 1]) with a given probability measure *P*(**s**):

4 $$\frac{dc(t,{\bf{s}})}{dt}=\rho c(t,{\bf{s}})(1-c(t,{\bf{s}}))(1-{\bf{s}})-k{\bf{s}}c(t,{\bf{s}})$$

5 $$c(t)={\int}_{\Omega }c(t,{\bf{s}})dP({\bf{s}})$$

where *c*(*t*) represents to total cell volume over time.

To explain the differential equation, we consider a pre-specified value of sensitivity *s*_*j*_. In the differential equation, we assume that both the growth and death terms will depend on sensitivity to treatment, and that the individual cell subpopulations do not compete with each other for space or resources. Hence, the equation describing cell density over time for a population that has sensitivity level *s*_*j*_ is given by:

6 $$\frac{dc}{dt}(t,{s}_{j})=\rho c(1-c)(1-{s}_{j})-k{s}_{j}c$$

7 $$c(0,{s}_{j})={c}_{0}.$$

Here, *ρ* is the maximal growth rate, *k* is the maximal death rate due to treatment, and *s*_*j*_ is sensitivity to treatment, restricted between 0 and 1. In most contexts, the maximal growth rate *ρ* will be smaller than the maximal death rate *k* since we assume that sensitive cells will die under treatment. We note that as *k* is scaled by the sensitivity, for cases in which the population is resistant (low values of sensitivity), the effective death rate of k*s_*j*_ will be smaller than the effective birth rate ρ*(1 − *s*_*j*_). The growth term, *ρc*(1 − *c*)(1 − *s*_*j*_), is logistic growth scaled by sensitivity to treatment in the form 1 − *s*_*j*_. When sensitivity to treatment is high (*s*_*j*_ values are closer to one), the growth is substantially slower, while when sensitivity to treatment is low (*s*_*j*_ values are closer to zero), growth is largely unimpeded by treatment. Meanwhile, the death term, − *ks*_*j*_*c*, contains *s*_*j*_ so that as sensitivity to treatment increases, so does the rate at which cells die. Please see Supplementary Note 1, Supplementary Fig. 8, and Supplementary Table 2 for information about equilibrium analysis.

In the case where the tumor is composed entirely of fully sensitive cells, *s*_*j*_ = 1, the differential equation loses the growth term entirely, leaving the population to die out over time in response to the treatment:

8 $$\frac{dc}{dt}=-kc$$

9 $$c(0)={c}_{0}.$$

Meanwhile, in the case where a tumor is composed entirely of fully resistant cells, *s*_*j*_ = 0, the differential equation loses the death term, meaning the tumor would continue to grow logistically:

10 $$\frac{dc}{dt}=\rho c(1-c)$$

11 $$c(0)={c}_{0}.$$

While the *s*_*j*_ = 0 and *s*_*j*_ = 1 cases are useful tools to reflect on what happens when a tumor is populated at the extreme ends of the sensitivity scale, they are not biologically likely to be the primary cell type in an overall tumor. As described in^1^, tumors being populated by a number of different cell types mean that different subpopulations will have different levels of sensitivity.

To mathematically solve Equation (5), we must make numerical approximations. First, we numerically discretize the compact probability space Ω = [0, 1]. We denote this numerical mesh *s*_*j*_, *j* = 1, . . . , *q*. Then, we approximate the PDF or PMF on that numerical mesh (denoted *p*(*s*_*j*_)). Next, we solve Equation (6) for each node in the *s* mesh, generating *c*(*t*; *s*_1_), *c*(*t*; *s*_2_), ..., *c*(*t*; *s*_*q*_). We then take *c*(*t*; *s*) as a weighted sum of each *c*(*t*; *s*_*j*_) curve, where the weights are the probability value *p*(*s*_*j*_) at each node of the sensitivity mesh *s*_*j*_. Hence, the total tumor *c*(*t*) is given by

12 $$c(t)=\mathop{\sum }\limits_{j=1}^{q}c(t,{s}_{j})p({s}_{j}),$$

where *q* is the number of points in the numerical mesh.

To test the inverse problem, we create synthetic data. It is necessary to choose parameter values for the sensitivity distribution, *ρ* and *k* values, and the time span. The sensitivity distribution is defined by choosing a continuous or discrete distribution from five options over some numerical sensitivity mesh, as described in the results section. For the numerical sensitivity mesh, s, we chose 100 mesh points to ensure smooth-looking distributions. The growth and death terms *ρ* and *k* are scalars of 0.3 and 0.45, respectively. These choices of *ρ* and *k* ensure that if cells have a sensitivity values of 1, the death rate will be larger than the growth rate. The time span can vary depending on the data, but is also defined using linspace with a mesh of 100 points.

In the context of tumor growth, where the error comes from attempting to count a large number of cells, the appropriate error is proportional to the size of the tumor. Hence, we create the simulated data by applying proportional error to the *c*(*t*) curve obtained by running the forward problem:

13 $$y(t)=c(t)+{n}_{l}\epsilon c(t)$$

where *ϵ* is independent and identically distributed via the standard normal distribution *N*(0, 1) and *n*_*l*_ represents the noise level, ranging from 0 to 0.05 (5% noise). Hence, *y*(*t*) depicts a tumor growing or shrinking over time with noise proportional to the number of cells.

We generated synthetic data for five separate underlying distributions, as described in the results section: one-point, two-point, uniform, Gaussian, and bi-Gaussian (see the results section for full details). The mesh for the underlying sensitivity distribution can be arbitrary, but we used a mesh of 100 points to ensure a smooth curve.

### Inverse problem methods

Given data describing how a tumor grows or shrinks over time, our goal is to recover the distribution of sensitivity to treatment. Ultimately, this means that we are attempting to approximate the probability measure *P*(**s**) as shown in Equation (2). To perform the inverse problem, we rely on the Prohorov Metric Framework, which has been developed over several decades^41,45,46^. The framework has been used in a wide variety of biological applications from protein aggregation^47^ to transdermal alcohol measurements^48^ to cancer growth and treatment^34,35,49^. For some tutorial and review papers on the method, see refs. ^41,50,51^. There are two established methods for estimating $\hat{P}$: a discrete method and a continuous method. The discrete estimation is appropriate for creating a PMF while the continuous estimation returns a PDF. In this manuscript, we only consider the discrete estimation, which returns a PMF.

Typically, available data is tumor size measured by counting cells or similar methods, meaning that the error is proportional to the data. Therefore, we use a generalized least squares method to minimize the sum of squared differences between the data and its approximation. We use Generalized Least Squares (GLS) as the framework for optimizing the recovered sensitivity distribution. GLS adapts the commonly used Ordinary Least Squares (OLS) method to proportional error, also called correlated error^52^. GLS and other very similar methods are commonly used, including in refs. ^34,37^. Banks et al.^40^ describe the generalized least squares estimate as

14 $$\hat{{\bf{P}}}=\mathop{{\rm{arg}}\, {\rm{min}} }\limits_{{\bf{s}}\in {\Omega }_{s}}\left(\mathop{\sum }\limits_{i=1}^{{N}_{t}}{w}_{i}^{-2}{({y}_{i}-f({t}_{i},{\bf{s}}))}^{2}\right),$$

where *f*(*t*_*i*_, **s**) is the solution to the forward problem defined in Eq. (6). Within the bounds of chosen tolerances, an initial value of **s,** $\hat{{\bf{s}}}$, is found using ordinary least squares (OLS). The weights *w*_*i*_ are computed using ${w}_{i}=f({t}_{i},\hat{{\bf{s}}})$ (the initial weights are set to $\frac{1}{{N}_{t}}$), and then $\hat{{\bf{s}}}$ is estimated again with the updated weights and $\hat{{\bf{s}}}$ estimated by Eq. (14). When an estimated $\hat{{\bf{s}}}$ is sufficiently close to the previous estimate, the loop concludes. Through this process, we minimize Eq. (15) to find the optimal sensitivity distribution for a given dataset.

Because the optimal sensitivity to treatment being recovered is a probability mass function, its values must be nonnegative and must sum to 1. Therefore, we aim to solve the following constrained optimization problem:

15 $$\,{\text{Minimize}}\,\mathop{\sum }\limits_{i=1}^{{N}_{t}}{w}_{i}^{-2}{\left(\mathop{\sum }\limits_{j = 1}^{q}c(i,{s}_{j})p({s}_{j})-{y}_{i}\right)}^{2}$$

16 $$\,{\rm{Subject}}\, {\rm{to}}\,\mathop{\sum }\limits_{j=1}^{q}p({s}_{j})=1$$

17 $$p({s}_{j})\ge 0\,\forall{j}$$

where *N*_*t*_ is the total number of time steps in the data, *w*_*i*_ is the weight for time step *i* found by GLS, *c*(*i*, *s*_*j*_) is the total tumor volume at time *i* found by solving Equation (6) for each *s*_*j*_ value in the *s* mesh, *p*(*s*_*j*_) is the proportion of population for each approximated *s*_*j*_ value, and *y*_*i*_ is the value of the data at the relevant time point. In the constraints, *q* is the number of points in the recovered sensitivity distribution.

The steps for performing the inverse problem for a given dataset to recover a probability mass function with a predetermined number of mesh points are as follows:

1. Numerically define the mesh for the recovered distribution. For a selected number of nodes, *q*, we numerically mesh the sensitivity values **s** on the interval [0,1] with *q* equidistant nodes.
2. Precompute the individual solutions to the forward problem (Equation (6)) for each sensitivity value *s*_*j*_, *j* = 1, . . . , *q* in the mesh.
3. Use constrained optimization to minimize the error function (Equation (15)) and uncover *p*(*s*_*j*_), *j* = 1, . . . , *q*, the optimal probability mass function. This is our estimate of the probability mass function, *P*(**s**).

We use Matlab’s FMINCON, a commonly used constrained optimization tool, to solve Eq. (15) as constrained by Eqs. (16) and (17). We chose to have FMINCON use a sequential quadratic programming (sqp) algorithm.

We can generate the CDF from the PMF and compute the aggregate tumor volume over time using the recovered PMF. However, while the datasets and model are supported by literature and reasoning, the numerical spacing of the mesh for the recovered distributions is not known a priori. Fortunately, we can try several different mesh densities and use the Akaike Information Criterion^53^ to choose the most optimal mesh.

### Akaike information criterion for optimal mesh selection

The inverse problem needs to be assigned a specific number of points for the distribution it recovers. However, there is no way to know a priori what number of points will yield the smallest error or the most relevant distribution. We use the Akaike Information Criterion to test a wide number of potential numerical meshes and select the most optimal recovered distribution. The Akaike Information Criterion (AIC) is a commonly used method for model selection^40,53,54^, weighing a reduced error against the cost of adding more parameter values. For the inverse problem, we use AIC to determine how many points are optimal in the distribution we are recovering, or in other words, the mesh for the sensitivity distribution.

Banks and Joyner^40^ describe AIC under a generalized least squares formation as

18 $$AI{C}_{GLS}={N}_{t}ln\left(\frac{\mathop{\sum }\nolimits_{i = 1}^{{N}_{t}}{w}_{i}^{-2}{\left(\mathop{\sum }\nolimits_{j = 1}^{q}c({t}_{i},{s}_{j})p({s}_{j})-{y}_{i}\right)}^{2}}{{N}_{t}}\right)+2(q+1),$$

where *i* is the index at each time step in the data, *N*_*t*_ is the number of time points, *w*_*i*_ is the weight derived by GLS, *j* is the index for the nodes of *s*, *y*_*i*_ is an observed data point at time *t*_*i*_, *t*_*i*_ is the time point, and *q* is the number of parameters in the model (e.g., the number of nodes in the recovered sensitivity distribution)^40^. As the number of parameters in the recovered distribution increases, the AIC score may increase or decrease depending on how the increased number of parameters affects the error. In this paper, we test over mesh sizes ranging from *q* = 4 to *q* = 30 in intervals of one. For an example of the AIC scores for the noiseless synthetic data case, please see Supplementary Fig. 2.

### Finding growth and death rates for in vitro data

The growth rate *ρ* and death rate *k* are not known a priori in the context of the in vitro monoclonal data. While^37^ provides a method for determining a net growth rate, it has limited application for Equation (6) because our model separately models growth and death rates. To separate the different subpopulations as clearly as possible, we need to structure the model such that when a population is completely sensitive to treatment, with *s* = 1, it will have the largest possible death rate. Similarly, a completely resistant population with *s* = 0 should have the largest growth rate. As a result, it is necessary to recover the maximum growth and death rates.

To find the maximum possible growth rate, ${\rho }_{\max }$, we use generalized least squares^40^ to fit the cell line data with no treatment administered. Each monoclonal and mixture dataset has one experiment in which no treatment is administered, and this is where we expect to find the fastest-growing curve. To find ${\rho }_{\max }$, we fit to the equation:

19 $$\frac{dc}{dt}=\rho c(1-c)$$

20 $$c(0)={c}_{0},$$

which is Equation (6) under the assumption of complete resistance (*s* = 0) or under the assumption of no treatment.

Similarly, to find the maximum possible death rate, we use the dataset that features the maximum decrease in tumor volume, and use generalized least squares to fit to the equation:

21 $$\frac{dc}{dt}=-kc$$

22 $$c(0)={c}_{0},$$

which is Eq. (6) with the assumption *s* = 1, or a completely sensitive population. Although we might reasonably expect that the maximal death rate would occur for a sensitive population at the highest dosage level, for one of the sensitive datasets, the second highest dosage level resulted in the largest estimate for ${k}_{\max }$.

**Fig. 9:**
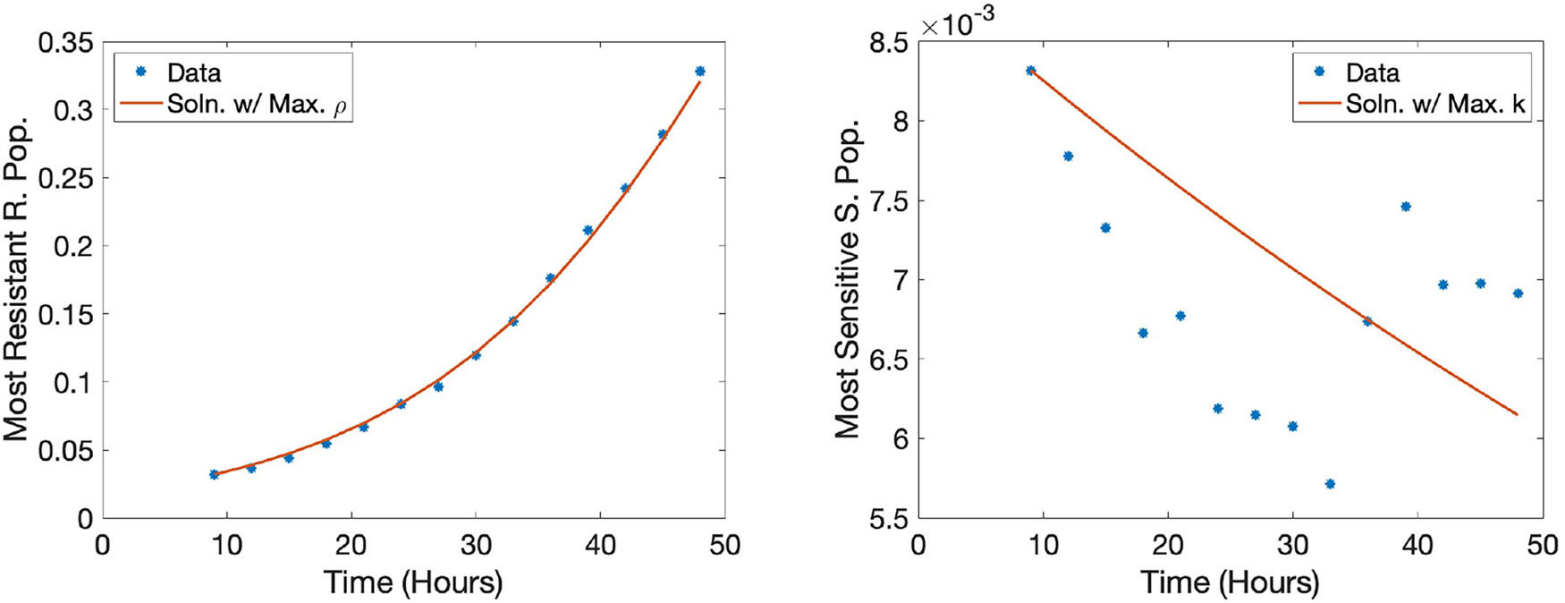
Estimates of *ρ* and *k*. The fits for maximum *ρ* found from the R500 dataset (left), as well as the fit for the maximum *k* found from the S1000 dataset (right).

Figure 9 displays the resulting best fit between data (blue circles) and model fit (red lines) for finding ${\rho }_{\max }$ (left) and ${k}_{\max }$ (right). It is clear that the fit for ${\rho }_{\max }$ is relatively good. The fit for ${k}_{\max }$ does not appear to be as well-explained by the model, in part due to the much smaller scale of the cell count and the resulting narrow y-axis. Table 2 displays the estimated parameter values for each of the datasets.

**Table 2:** Recovered maximum *ρ* values from the resistant datasets and recovered maximum *k* values from the sensitive datasets

Dataset	Max. *ρ*	Max. *k*
R250	0.0692	n/a
R500	0.0682	n/a
S500	n/a	0.0019
S1000	n/a	0.0077

After obtaining the scalar values for the maximum *ρ* and *k*, it is necessary to adjust death rate *k* by dosage for each concentration level in the dataset (the vector of dosage concentrations is depicted in Table 1). We scale *k* for each concentration according to dosage level. We make the following assumptions: (1) For completely resistant data, tumor growth would not depend on dosage because completely resistant cells would be immune to any dosage. Hence, the growth rate will remain constant across dosage. (2) Because sensitive cells die at different rates depending on dosage, the death rate will increase as dosage increases. (3) The minimal death rate is 0 and occurs when no treatment is applied (e.g., dosage is 0). Thus, we scale the constant *k* values using

23 $$k(m)={k}_{\max }\left(\frac{d(m)}{{d}_{\max }}\right)$$

for each concentration number *m* of dosage *d*, where ${d}_{\max }$ represents the maximum dosage value. Hence, the forward problem will use a different death rate for each different drug concentration, while the growth rate will not depend on drug concentration.

While it is possible to uncover the maximum growth rates for the resistant monoclonal data and the maximum death rate for the sensitive monoclonal data, there is no such clear process to estimate the growth and death rates for the mixture data. To approximate usable growth and death rates, we chose the larger of the two maximum growth rates and the larger of the two maximum death rates from Equation 2, reasoning that given the ratios of sensitive to resistant cells, growth and death rates close to the absolute maximum would offer enough flexibility to work well with the mixture data. Hence, for the mixture data, *ρ* = 0.0692 and *k* = 0.0077.

## Supplementary information

Supplementary Material

## Data Availability

The synthetic data generated for this study is available on GitHub and can be accessed via this link https://github.com/NatalieMeacham/synthetic-and-in-vitro-inverse-problem. The in vitro monoclonal and mixture data are available in the Phenopop paper^37^ and their associated GitHub repository.

## Code availability

The underlying code for this study is available on GitHub and can be accessed via this link https://github.com/NatalieMeacham/synthetic-and-in-vitro-inverse-problem.
