# Supplementary material for: Estimating treatment sensitivity in synthetic and in vitro tumors using a random differential equation model
Source: NPJ Syst Biol Appl. 2025 May 23;11:54. doi: 10.1038/s41540-025-00530-0 (PMC12102228; doi:10.1038/s41540-025-00530-0)
Supplement: Supplementary file 1 — Supplementary Material [file 41540_2025_530_MOESM1_ESM.pdf]

Supplementary Material for "Estimating Treatment Sensitivity in Synthetic and *In Vitro* Tumors Using a Random Differential Equation Model"

Natalie Meacham and Erica M. Rutter

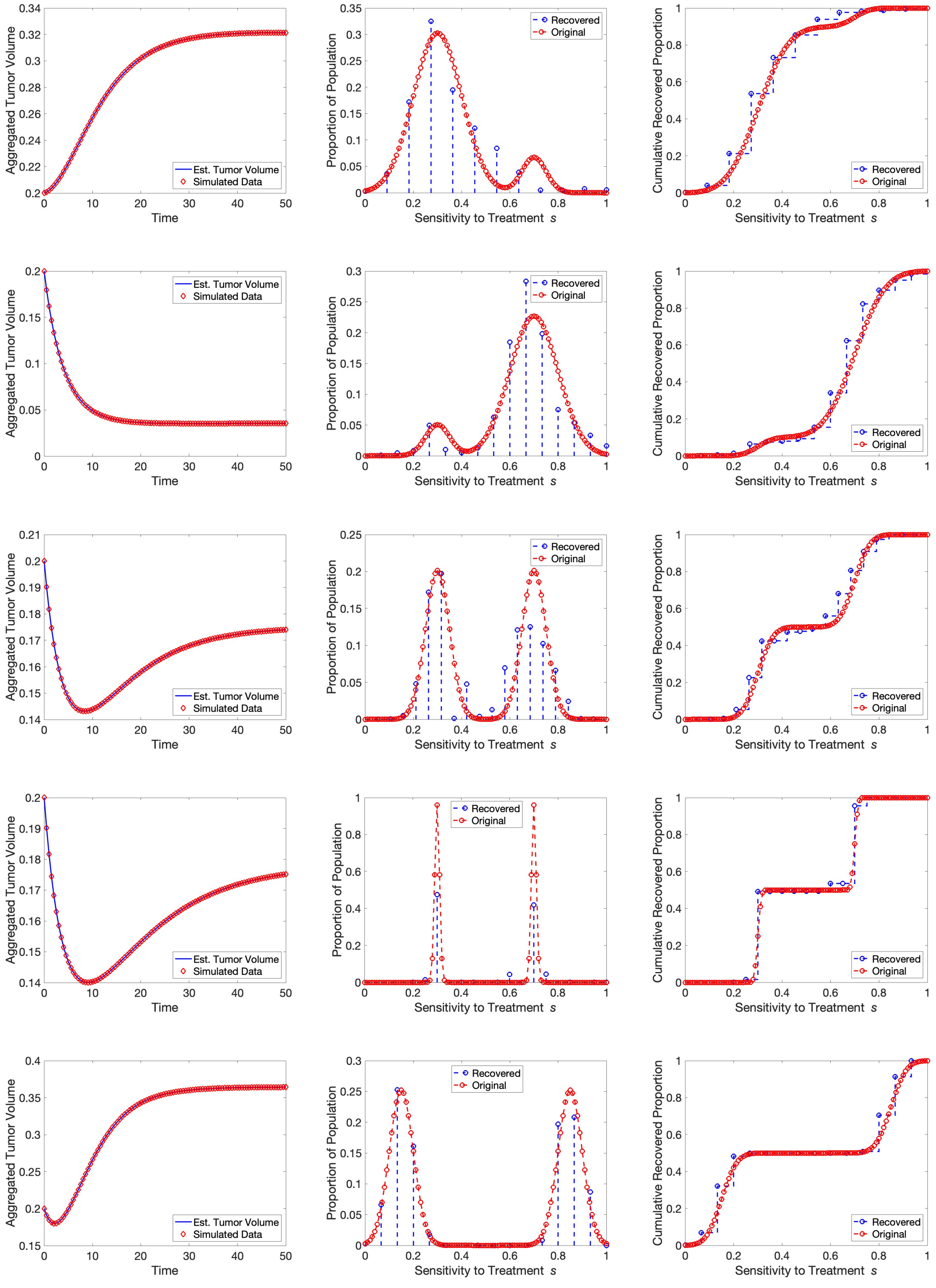

Supplementary Figure 1: Results for a variety of bi-Gaussian distributions. Each row represents a different true underlying bi-Gaussian distribution (details in Supplementary Table 1). Left panels display tumor volume fits (original in red circles, recovered in blue circles), middle panels depict recoverd PMFs (blue) versus true underling PDF/PMF (red) and right panels contain the underlying and predicted CDFs.

Row	μ_1	σ_1	Weight 1	μ_2	σ_2	Weight 2
1	0.3	0.1	0.9	0.7	0.05	0.1
2	0.3	0.05	0.1	0.7	0.1	0.9
3	0.3	0.05	0.5	0.7	0.05	0.5
4	0.3	0.01	0.5	0.7	0.01	0.5
5	0.15	0.05	0.5	0.85	0.05	0.5

Supplementary Table 1: Information about the bi-Gaussian distributions plotted in Supplementary Figure 1.

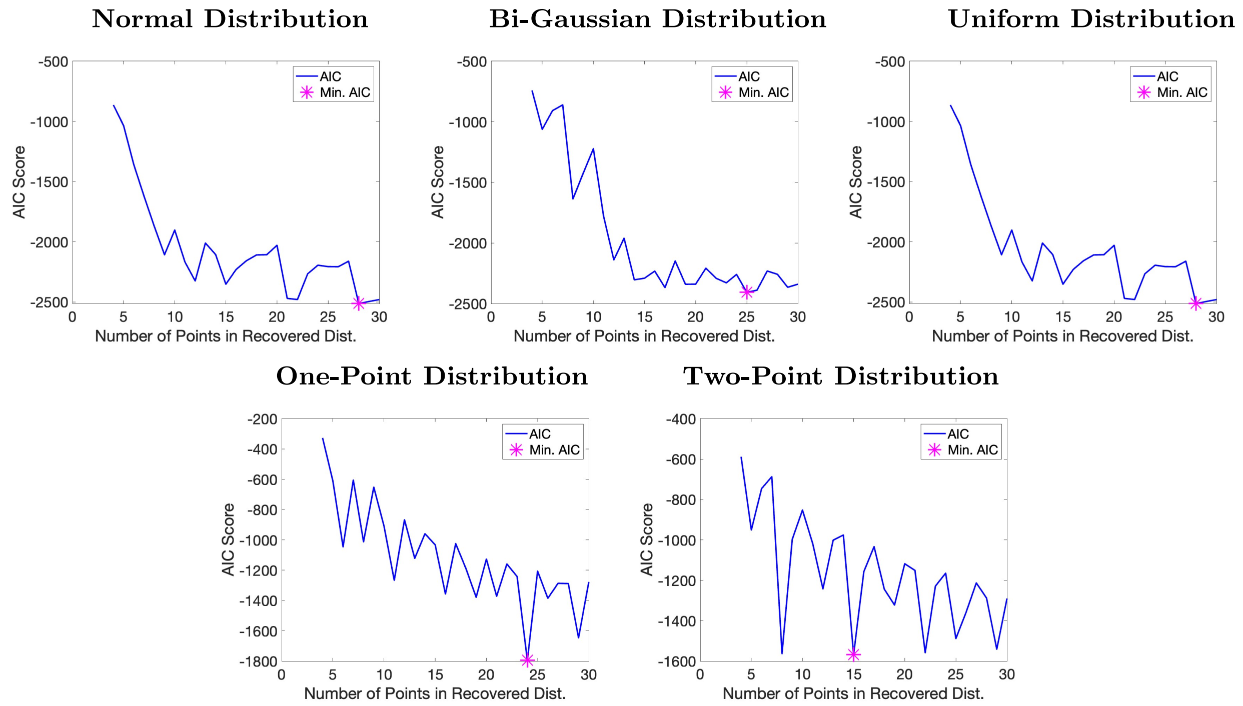

Supplementary Figure 2: Akaike Information Criterion (AIC) values across different meshes (e.g., number of parameters) for the five synthetic underlying distributions in the case of no added noise. Minimal AIC value is highlighed in pink asterisk.

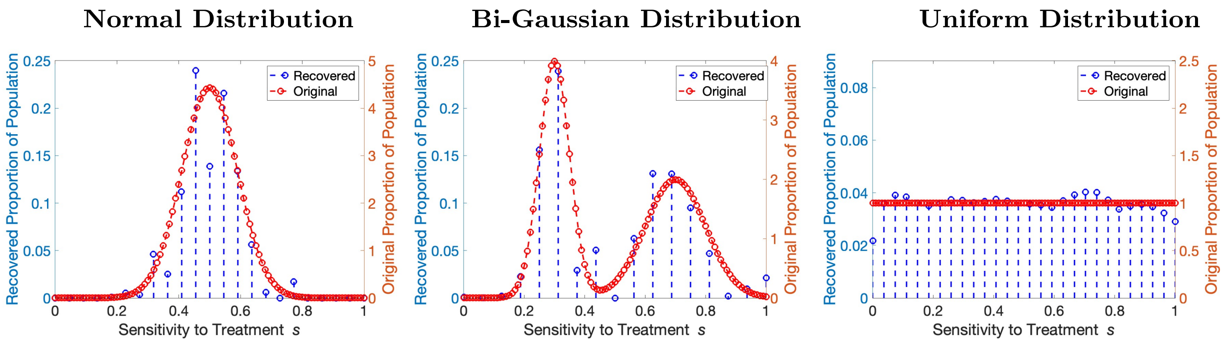

Supplementary Figure 3: Raw recovered PMF results for continuous underlying distributions. Original distributions in red (and right axis) with recovered distributions in blue (left axis). Axis limits were selected to allow a similar comparison between recovered and original distributions. Recovered PMFs were not rescaled in order to match underlying PDFs.

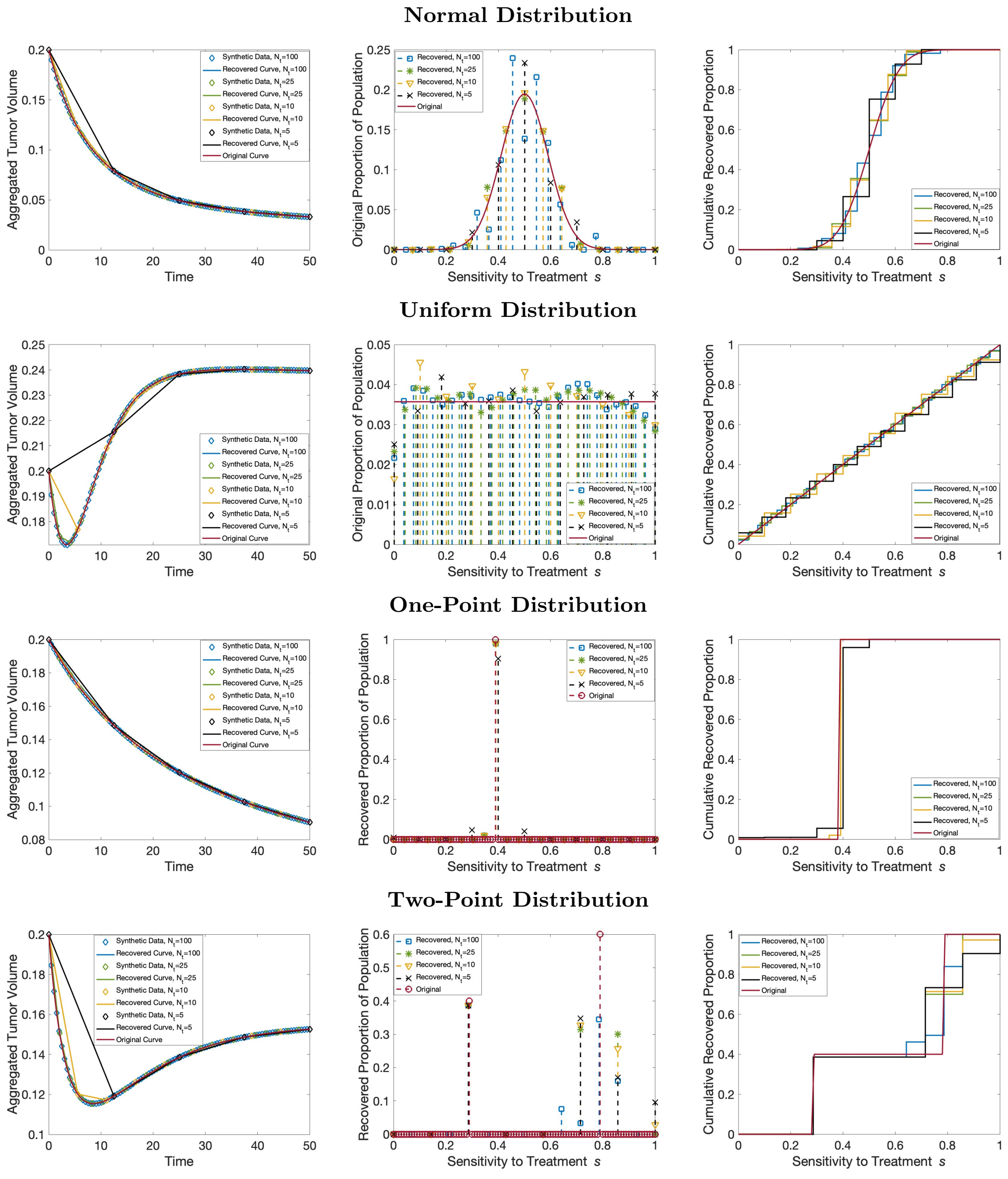

Supplementary Figure 4: Results for inverse problem on synthetic data decreasing timepoints. Resulting tumor volume fits (left), recovered PMFs (middle) and CDFs (right) assuming 100 data points (blue squares), 25 time points (green asterisks), 10 time points (yellow triangles), and 5 time points (black x's) for the Gaussian, uniform, one-point, and two-point distributions.

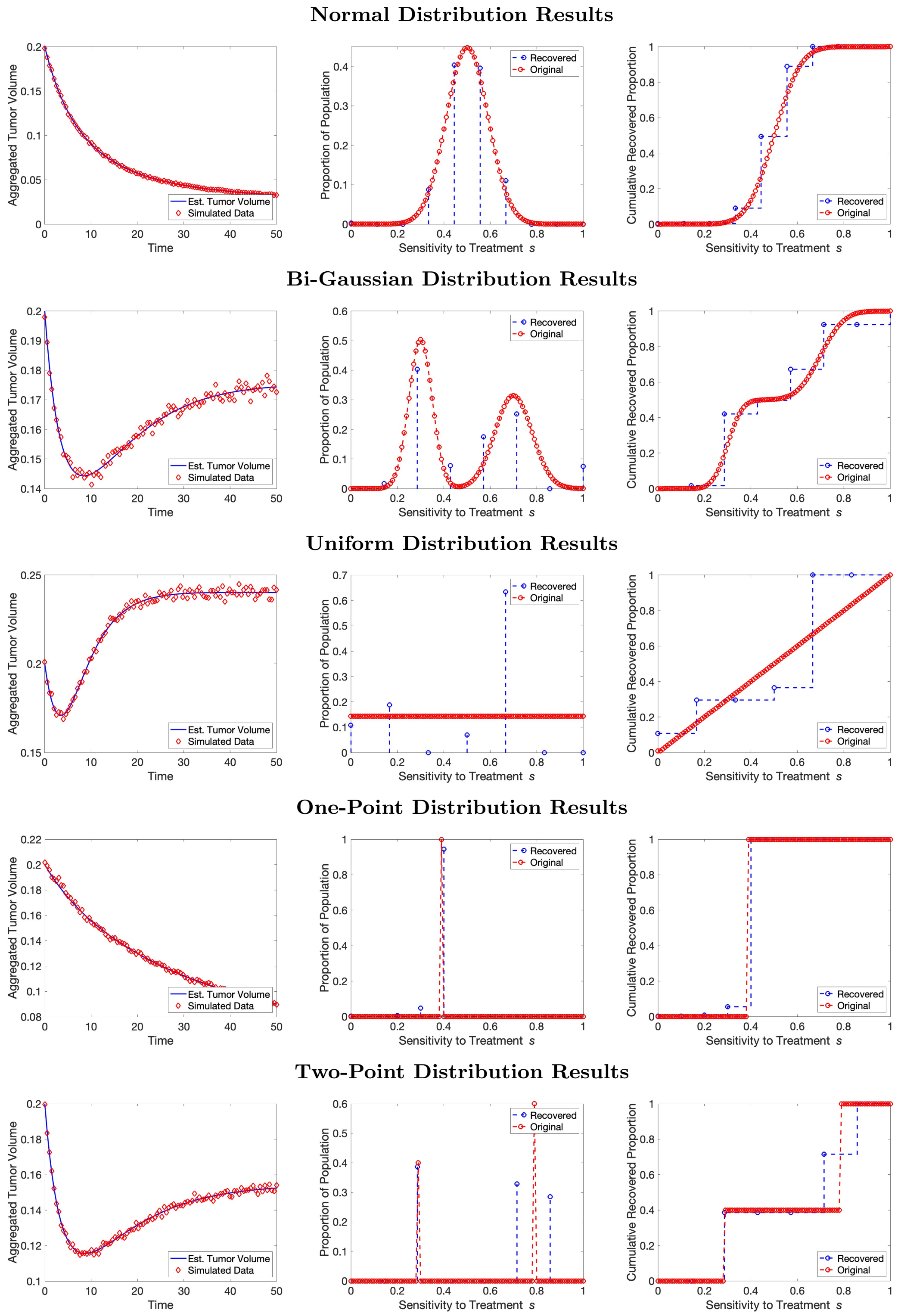

Supplementary Figure 5: Recovered fits (left), PMFs (middle), and CDFs (right) for five distributions with 1% proportional noise. A small level of noise allows for close fits to the data and recovered PMFs that closely resemble the originals, with the exception of the uniform distribution.

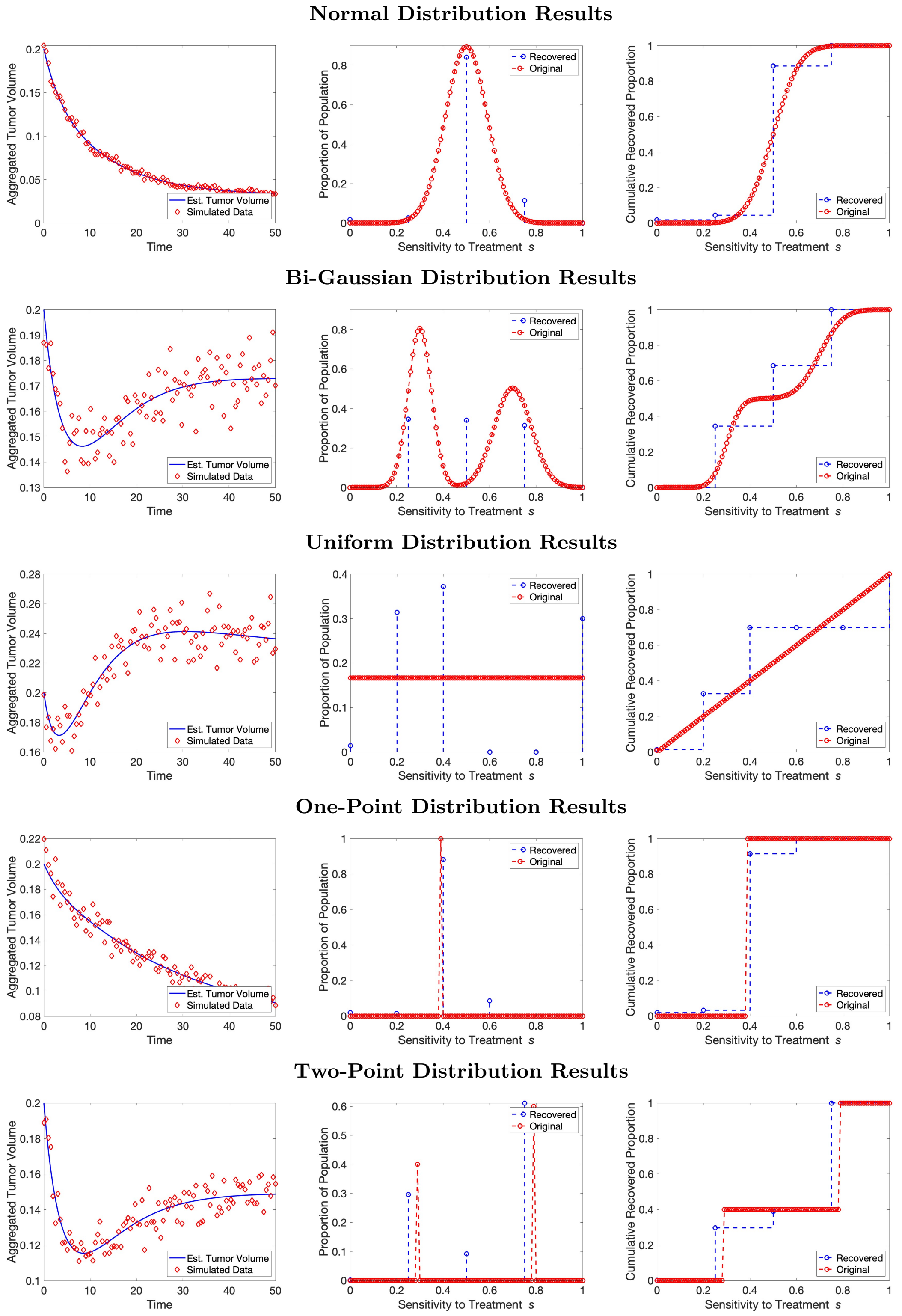

Supplementary Figure 6: Recovered fits (left), PMFs (middle), and CDFs (right) for five distributions with 5% proportional noise. At this higher level of noise, the data fit is still successful but has higher error, and the PMFs are recovered much more vaguely.

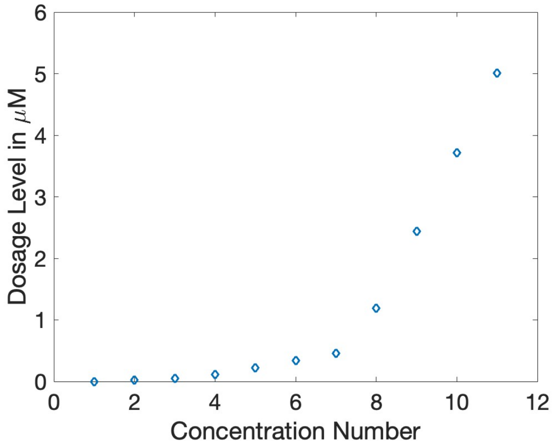

Supplementary Figure 7: Drug concentration administered for each of the 11 dosages in the monoclonal and mixture *in vitro* datasets [1].

Supplementary Note 1: Equilibrium Analysis

To understand long-term behavior of solutions, we analyze the equilibrium points and assess their stability under the assumption that s is a fixed constant. We briefly list the model below:

$$\frac{dc}{dt}(t, s_j) = \rho c(1-c)(1-s_j) - k s_j c \tag{1}$$

$$c(0, s_j) = c_0. \tag{2}$$

Theorem 1. *The model Equation (1) has a disease-free equilibrium $c_0^* = 0$ that is locally asymptotically stable when $s > \frac{\rho}{\rho+k}$ and unstable when $s < \frac{\rho}{\rho+k}$. When $s < \frac{\rho}{\rho+k}$, a stable positive endemic equilibrium, $c_1^* = \frac{\rho s - \rho + ks}{\rho s - \rho}$, emerges.*

Proof. We solve for the equilibrium points by setting Equation (1) to zero and solving for c^*:

$$0 = \rho c^*(1-c^*)(1-s) - ksc^* \tag{3}$$

$$0 = c^*[\rho(1-c^*)(1-s) - ks] \tag{4}$$

After simplifying, the two equilibria for Equation (1) are

$$c_0^* = 0 \tag{5}$$

$$c_1^* = \frac{\rho s - \rho + ks}{\rho s - \rho}. \tag{6}$$

For $c_1^* > 0$, it is necessary to have $s \leq \frac{\rho}{\rho+k}$. Hence, there will be a negative equilibrium when $s > \frac{\rho}{\rho+k}$.

To determine the stability of the equilibria, we differentiate Equation (1) and plug in each of the two equilibria:

$$\frac{d}{dc}\left(\frac{dc}{dt}\right) = \rho(1-s)(1-2c) - ks \tag{7}$$

For $c_0^* = 0$, this simplifies to $\frac{d^2c}{dt^2}(c_0^*) = \rho - \rho s - ks$. Thus, when $s < \frac{\rho}{\rho+k}$, the zero equilibrium is unstable, and when $s > \frac{\rho}{\rho+k}$, the zero equilibrium is stable.

For $c_1^* = \frac{\rho s - \rho + ks}{\rho s - \rho}$, this simplifies to $\frac{d^2c}{dt^2}(c_1^*) = \rho s - \rho + ks$. Thus, when $s < \frac{\rho}{\rho+k}$, this equilibrium is stable, and when $s > \frac{\rho}{\rho+k}$, the equilibrium becomes unstable (and also negative).

□

At $s = \frac{\rho}{\rho+k}$, there is a transcritical bifurcation where the model switches from a stable positive equilibrium and an unstable zero equilibrium to an unstable negative equilibrium and a stable zero equilibrium. In terms of the biology of the model, this means that cells that have low sensitivity will have a positive stable equilibrium (e.g., the tumor will grow). For cells that have high sensitivity, the zero equilibrium will be stable (e.g., tumor will die out). This information is summarized in Supplementary Table 2. Additionally, Supplementary Figure 8 depicts the bifurcation diagram for the k and ρ values used for the synthetic data in the main manuscript.

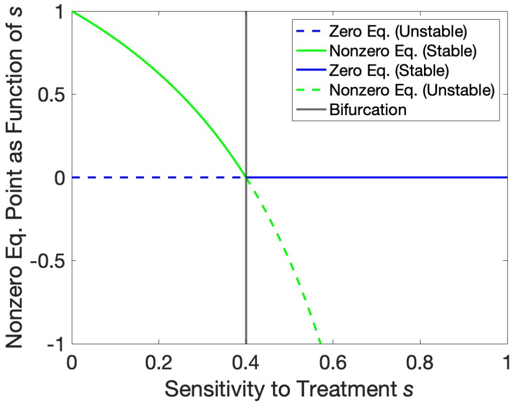

Supplementary Figure 8: As sensitivity to treatment s increases, a transcritical bifurcation occurs at $s = \frac{\rho}{\rho+k}$ for $\rho = 0.3$ and $k = 0.45$.

Stability Condition	Zero Eq.	Nonzero Eq.
$s > \frac{\rho}{\rho+k}$	stable	negative, unstable
$s < \frac{\rho}{\rho+k}$	unstable	positive, stable

Supplementary Table 2: Equilibria descriptions for each stability condition for Equation (1).

References

[1] Alvaro Köhn-Luque, Even Moa Myklebust, Dagim Shiferaw Tadele, Mariaserena Giliberto, Leonard Schmiester, Jasmine Noory, Elise Harivel, Polina Arsenteva, Shannon M Mumenthaler, Fredrik Schjesvold, et al. Phenotypic deconvolution in heterogeneous cancer cell populations using drug-screening data. *Cell Reports Methods*, 3(3), 2023.
